# A lateral flow immunoassay for the rapid identification of *Candida auris* from isolates or directly from surveillance enrichment broths

**DOI:** 10.3389/fmicb.2024.1439273

**Published:** 2024-07-03

**Authors:** Arnaud Chalin, Antoine Arvor, Anne-Sophie Hervault, Marc Plaisance, Léa Niol, Stéphanie Simon, Hervé Volland

**Affiliations:** ^1^NG Biotech – Research and Development Department, Guipry-Messac, France; ^2^Université Paris-Saclay, Commissariat à l'Energie Atomique et aux Energies Alternatives (CEA), Institut National de Recherche pour l'Agriculture, l'Alimentation et l'Environnement (INRAE), Médicaments et Technologies pour la Santé (MTS), Service de Pharmacologie et d'Immunoanalyse (SPI), Laboratoire d'Etudes et de Recherches en Immunoanalyse (LERI), Gif-sur-Yvette, France

**Keywords:** *Candida auris*, lateral flow immunoassay, surveillance sample, identification, validation

## Abstract

**Introduction:**

*Candida auris* is a recently discovered yeast with a multi-drug resistant profile associated with high mortality rates. The rapid identification of *Candida auris* in hospital settings is crucial to allow appropriate therapeutic and rapid implementation of infection management measures. The aim of this study was to develop a lateral flow immunoassay (LFIA) for the rapid identification of *Candida auris*.

**Methods:**

Highly specific monoclonal antibodies were obtained by immunizing mice with membrane proteins from *Candida auris* which were then used to develop a LFIA whose performance was assessed by testing 12 strains of *Candida auris* and 37 strains of other Candida species. Isolates were grown on either Sabouraud dextrose, CHROMagar^TM^ Candida Plus or HardyCHROM^TM^ *Candida* + *auris* agar plates. The strains were also cultured on salt sabouraud-dextrose with chloramphenicol or a commercially available Salt-Sabouraud Dulcitol Broth with chloramphenicol and gentamicin, and processed using a simple centrifugation protocol to recover a pellet. Finally, the colonies or yeast extract were transferred to the LFIA to determine the specificity and sensitivity of the assay.

**Results:**

The LFIA reached 100% specificity and sensitivity from solid agar plates. For both enrichment broths, some Candida non-auris species were able to grow, but the LFIA remained 100% specific. The use of a dextrose-based sabouraud broth resulted in earlier identification with the LFIA, with most of the Candida auris strains detected at 24 h.

**Conclusion:**

The developed LFIA prototype represents a powerful tool to fight the emerging threat of Candida auris. Clinical validation represents the next step.

## 1 Introduction

Nosocomial candidiasis represents a major threat to immunocompromised and critically-ill patients, and is the most important fungal infection in hospitalized patients worldwide (Brown et al., 2012; Pfaller and Castanheira, 2016). The mortality rate of candidemia, a life-threatening condition, has not decreased over the past two decades despite the introduction of new antifungal agents (Pfaller and Castanheira, 2016). This is a result of the complexity of *Candida* infection diagnosis, leading to challenges in rapid administration of adequate antifungal therapy, and inappropriate use of empiric therapies (Garey et al., 2006; Azoulay et al., 2012; Drgona et al., 2014).

Among the *Candida* species, *Candida auris* is a recently discovered ascomycetous yeast first identified in 2009 from the external ear canal of an inpatient in a Japanese hospital (Satoh et al., 2009). *Candida auris* simultaneously emerged on different continents around the world (Lockhart et al., 2017) and is currently divided into four major clades plus 1 minor clade (Lockhart et al., 2017; Chow et al., 2019). The rapid spread of *Candida auris*, associated high mortality rates (30–72%) (Chakrabarti et al., 2015; Lockhart et al., 2017; Jeffery-Smith et al., 2018; Ruiz-Gaitan et al., 2018; Osei Sekyere, 2019) and its multi-drug resistant profile justify the attention being paid to this worldwide emerging threat (Chowdhary et al., 2017; WHO, 2022). Indeed, *Candida auris* is usually resistant to the azole antifungal class, including fluconazole and voriconazole, and can also show resistance to echinocandins, amphotericin B and flucytosine (Ruiz Gaitan et al., 2017; Jacobs et al., 2022; Kilburn et al., 2022). Moreover, it is very worrying to note the emergence of pan-resistance to four major classes of antifungals in some *Candida auris* clinical isolates (Jacobs et al., 2022).

Risk factors are identical to those of candidiasis caused by other species and typical of opportunistic and nosocomial organisms, namely: prolonged hospitalization especially in an intensive care unit (ICU) (Rudramurthy et al., 2017), and immunosuppression or prior exposure to broad-spectrum antibiotics and antifungal therapy (Calvo et al., 2016; Lockhart et al., 2017; Rudramurthy et al., 2017). *Candida auris* is a notable nosocomial agent due to its opportunistic behavior, but also because of its persistence on surfaces (Welsh et al., 2017; Short et al., 2019) and ability to colonize patient skin (Horton et al., 2020; Uppuluri, 2020). Consequently, *Candida auris* can easily spread in the hospital environment if infection control measures are insufficient, including disinfection, isolation and testing protocols (Eckbo et al., 2021; Prestel et al., 2021; Villanueva-Lozano et al., 2021; Thoma et al., 2022).

The rapid identification of *Candida auris* in hospital settings is therefore crucial to avoid therapeutic failure, fatal outcomes, to limit selective pressure caused by antifungal misuse and to allow infection management measures to be implemented as soon as possible.

*Candida auris* colonization can be determined by taking axilla or groin swabs, followed by an enrichment step using Sabouraud-based broth containing 10% sodium chloride and incubation for several days (Welsh et al., 2017). This method can be used to selectively look for *Candida auris* colonization, but still requires the yeast to be isolated on a solid media (Sabouraud or specific chromogenic media) for further examination, which requires a further 48 h or more until final identification. In the past, *Candida auris* has been misidentified as other *Candida* or yeast species (Lone and Ahmad, 2019) illustrating the need for new and updated identification methods. In recent years, substantial work has been performed in this direction with the development of selective and differential chromogenic culture media, several Deoxyribonucleic Acid (DNA)-based diagnostics, biochemical assimilations and protein profiles (Dennis et al., 2021). However, these are expensive, require trained technicians as well as complex equipment, thus limiting their broad availability. Affordable and easy-to-use tools are still required to fight effectively the spread of *Candida auris* in hospital settings (Dennis et al., 2021). Lateral flow immunoassay (LFIA) technology is fast, affordable, easy-to-use and highly suitable for use in hospital laboratories, but also in frontline and low-resource laboratories. In the last decade, LFIA tests have become key tools in the fight against antimicrobial resistance (AMR) (Boutal et al., 2022) among others. The aim of this work was to select, characterize and produce monoclonal antibodies (Mabs) specific for *Candida auris* and develop a LFIA prototype for the rapid identification of *Candida auris* on agar plates or directly from cloudy enrichment broths.

## 2 Materials and methods

### 2.1 Ethics statements

All experiments were performed in compliance with French and European regulations on the care of laboratory animals (European Community Directive 86/609, French Law 2001-486, 6 June 2001) and with the agreements of the Ethics Committee of the Commissariat à l'Energie Atomique (CEtEA “Comité d'Ethique en Experimentation Animale” n°44) nos. 12-026 and 15-055 delivered to Stéphanie Simon by the French Veterinary Services and CEA agreement D-91-272-106 from the Veterinary Inspection Department of Essonne (France).

### 2.2 Reagents

Phosphate-buffered saline (PBS) (ref. 524650), β-mercaptoethanol (ref. 63689, CAS: 60-24-2), sodium chloride (NaCl) (ref. 31434, CAS: 7646-14-5), chloramphenicol (ref. C0857, CAS: 56-75-7) and NHS-biotin (ref. B2643, CAS: 72040-63-2) were provided by Sigma Aldrich (Saint Quentin Fallavier, France). Sabouraud dextrose medium (2% dextrose and 1% peptone digest) was either provided by Sigma Aldrich (ref. S3306) or Beckton Dickinson (ref. 215193). All the *Candida* strains used are listed in Table 1. Biozzi mice were bred at the animal care unit of CEA (Gif sur Yvette, France). Sabouraud dextrose agar plates were purchased from Liofilchem (ref. 10035), CHROMagar^TM^ *Candida* Plus manufactured by CHROMagar^TM^, and HardyCHROM^TM^ *Candida* + auris manufactured by HardyCHROM^TM^. Sabouraud-Salt Dulcitol Broth (SSDB) supplemented with chloramphenicol and gentamicin was manufactured by S2Media^TM^ (ref. 5137) and was made according to the Centre for Disease Control (CDC) formula recommendation for isolation and enrichment of *Candida auris*. Enzyme immunoassays (EIAs) were performed with Maxisorp 96-well microtiter plates (Nunc) (Paris, France), and all reagents were diluted in EIA buffer [0.1 M phosphate buffer pH 7.4 containing 0.15 M NaCl, 0.1% bovine serum albumin (BSA) and 0.01% sodium azide]. Plates coated with proteins were saturated in EIA buffer (18 h at 4°C) and washed with washing buffer (0.1 M potassium phosphate pH 7.4 containing 0.05% Tween 20). Plates coated with yeasts were saturated in EIA buffer (18 h at 4°C) and washed with washing buffer without Tween^®^ 20 (0.1 M potassium phosphate pH 7.4). Nitrocellulose strips with polystyrene backing were from GE Healthcare (Prima 40). Ellman's medium [7.5 × 10^−4^ M acetylthiocholine iodide (enzyme substrate) and 2.5 × 10^−4^ M 5,5′-dithiobis 2 nitrobenzoic acid (DTNB)], streptavidin-acetylcholinesterase (G4), Goat anti-mouse antibody-acétylcholinesterase and phosphate buffered saline pH 7.4 (10 mM sodium phosphate pH 7.4 containing 150 mM NaCl) were prepared at CEA (Gif-sur-Yvette, France). BCA protein assay kit (ref. 23235) was provided by Thermo Scientific™ (Waltham, Massachusetts, USA). Goat anti-mouse IgG/IgM polyclonal antibodies were provided by Jackson ImmunoResearch (ref. 115-005-044, Ely, United Kingdom). The colloidal gold solution was provided by NG Biotech (Guipry, France).

**Table 1:** List of *Candida auris* and non-*auris* strains used for immunization of mice, selection of the best monoclonal antibodies and/or validation of the lateral flow immunoassay prototype.

**Strain**	**Source**	**Used for**	**Clade**
*Candida auris* (#8971)	NCPF	Immunization and selection	South Asian
*Candida auris* (#8977)	NCPF	Immunization and selection	South African
*Candida auris* (#8984)	NCPF	Immunization and selection	East Asian
*Candida auris* (Z485)	Zeptometrix	Validation	Unknown
*Candida auris* (105991/CDC AR-Bank 0386)	DSMZ	Validation	South America
*Candida auris* (105989/CDC AR-Bank 0384)	DSMZ	Validation	South African
*Candida auris* (105990/CDC AR-Bank 0385)	DSMZ	Validation	South American
*Candida auris* (105988/CDC AR-Bank 0383)	DSMZ	Validation	South African
*Candida auris* (105987/CDC AR-Bank 0382)	DSMZ	Validation	South Asian
*Candida auris* (105992/CDC AR-Bank 0387)	DSMZ	Validation	South Asian
*Candida auris* (21092)	DSMZ	Validation	East Asian
*Candida auris* (NR-52714)	BEI resources	Validation	South Asian
*Candida auris* (NR-52715)	BEI resources	Validation	South Asian
*Candida auris* (NR-52716)	BEI resources	Validation	South Asian
*Candida auris* (NR-52717)	BEI resources	Validation	South Asian
*Candida haemulonii* (#8402)	NCPF	Selection	–
*Candida albicans* (14053)	ATCC	Validation	–
*Candida albicans* (18804)	ATCC	Validation	–
*Candida albicans* (64124)	ATCC	Validation	–
*Candida albicans* (Z006)	Zeptometrix	Validation	–
*Candida albicans* (NR-29342)	BEI resources	Validation	–
*Candida albicans* (NR-29343)	BEI resources	Validation	–
*Candida albicans* (NR-29344)	BEI resources	Validation	–
*Candida albicans* (NR-29452)	BEI resources	Validation	–
*Candida albicans* (NR-29453)	BEI resources	Validation	–
*Candida glabrata* (2001)	ATCC	Validation	–
*Candida glabrata* (15126)	ATCC	Validation	–
*Candida glabrata* (66032)	ATCC	Validation	–
*Candida glabrata* (Z007)	Zeptometrix	Validation	–
*Candida glabrata* (HM-1123)	BEI resources	Validation	–
*Candida krusei* (14243)	ATCC	Validation	–
*Candida krusei* (34135)	ATCC	Validation	–
*Candida krusei* (Z009)	Zeptometrix	Validation	–
*Candida krusei* (HM-1122)	BEI resources	Validation	–
*Candida parapsilosis* (22019)	ATCC	Validation	–
*Candida parapsilosis* (Z011)	Zeptometrix	Validation	–
*Candida tropicalis* (1369)	ATCC	Validation	–
*Candida tropicalis* (13803)	ATCC	Validation	–
*Candida tropicalis* (66029)	ATCC	Validation	–
*Candida tropicalis* (Z012)	Zeptometrix	Validation	–
*Candida tropicalis* (HM-1124)	BEI resources	Validation	–
*Candida guilliermondii* (Z008)	Zeptometrix	Validation	–
*Candida lusitaniae* (Z010)	Zeptometrix	Validation	–
*Candida sojae* (Z128)	Zeptometrix	Validation	–
*Candida kefyr* (Z110)	Zeptometrix	Validation	–
*Candida dubliniensis* (Z145)	Zeptometrix	Validation	–
*Candida catenulate* (Z253)	Zeptometrix	Validation	–
*Candida duobushaemulonii* (105996/CDC AR-Bank 0391)	DSMZ	Validation	–
*Candida duobushaemulonii* (105997/CDC AR-Bank 0392)	DSMZ	Validation	–
*Candida duobushaemulonii* (105999/CDC AR-Bank 0397)	DSMZ	Validation	–
*Candida haemulonii* (70624)	DSMZ	Validation	–
*Candida haemulonii* (105998/CDC AR-Bank 0393)	DSMZ	Validation	–

NCPF, National Collection of Pathogenic Fungi; DSMZ, German Collection of Microorganisms and Cell Cultures; ATCC, American Type Culture Collection; CDC, Center for Disease Control.

### 2.3 Preparation of immunogen and biotinylated surface proteins

Three *Candida auris* strains (#8971, #8977, #8984) (Table 1) were used for mice immunization and antibody selection. One *Candida haemulonii* strain (#8402) was used for antibody selection. *Candida* species were routinely maintained either in liquid Sabouraud dextrose medium, or on Sabouraud dextrose agar plates. For long-term storage, *Candida* cultures were kept frozen at −80°C with 20% glycerol. Using a 1 μL inoculation loop, yeasts in glycerol stock solution were transferred to a small volume of Sabouraud dextrose medium. Overnight grown yeasts (37, 35, or 30°C for *Candida auris, Candida haemulonii* and *Candida albicans*, respectively) were then used to inoculate a larger volume of Sabouraud dextrose medium. Yeast cells were pelleted twice by centrifugation at 3,000 g for 10 min and resuspended in the same volume of phosphate buffered saline pH 7.4 for washing. After the last wash, yeasts were resuspended in one tenth of the initial culture volume in ammonium carbonate buffer (1.89 g/L) containing 1% β-mercaptoethanol and incubated at 37°C for 30 min as described by Casanova and Chaffin (1991) for surface protein shaving. Supernatant was then collected by centrifugation at 3,000 g for 10 min and filtered at 0.22 μm to remove remaining yeast cells and before being dialysed thoroughly at 4°C in a capped Becher against phosphate buffered saline pH 7.4 to remove β-mercaptoethanol. Protein concentration was assessed with a BCA protein assay. Surface proteins were aliquoted and stored at −20°C until further use.

The immunogen preparation was made by mixing equivalent proportions of the surface proteins from the 3 *Candida auris* strains at 500 μg/mL in 50 mM potassium phosphate buffer pH 7.4. The surface protein solution was then mixed equally with Alum adjuvant to obtain the immunogen preparation.

Finally, the biotinylated surface proteins were prepared as follows. The medium protein size in the mix was approximately estimated by running surface proteins on SDS-PAGE. Biotin was conjugated to surface proteins by mixing NHS-biotin with surface proteins in 0.1 M borate buffer pH 9.0 (ratio NHS-biotin/protein = 20) and incubated for 30 min at room temperature. Unreacted ester functions were inactivated with 1 M Tris buffer pH 8.0 for 15 min at room temperature. Biotinylated surface proteins were then diluted in EIA buffer.

### 2.4 Production and screening of mouse monoclonal antibodies

**Figure 1:**
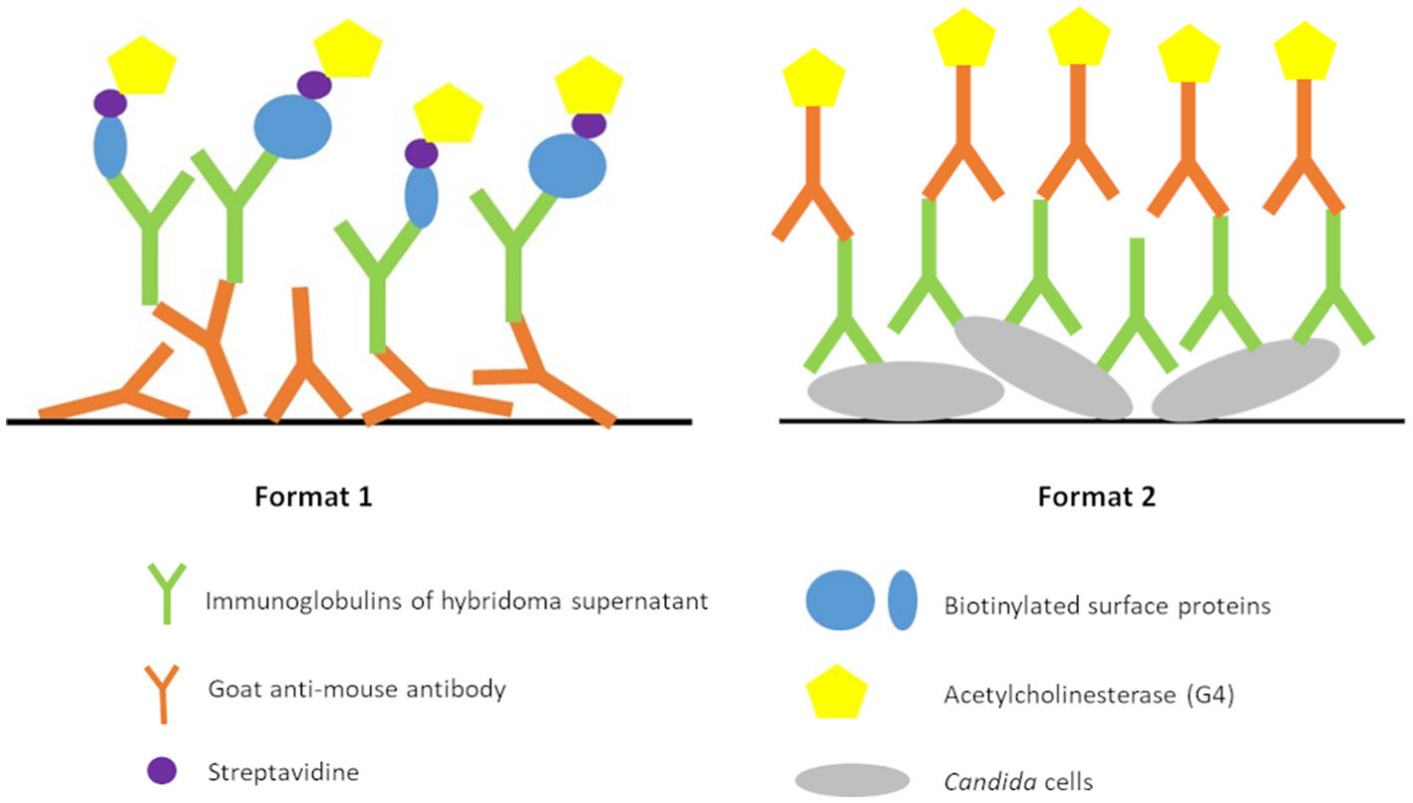
Immunoenzymatic test formats used for hybridoma screening.

Ten-week old Biozzi mice were immunized every 3 weeks with 50 μg of immunogen by intraperitoneal injection. Mice were bled to recover sera, before the first immunization and 2 weeks after each immunization. The polyclonal response was evaluated by testing sera with an immunoenzymatic test. Briefly, in microtiter plates coated with goat anti-mouse IgG/IgM polyclonal antibodies, 50 μl of biotinylated surface proteins were added to 50 μl of each serum diluted in EIA buffer. After overnight incubation at 4°C, the plates were washed with washing buffer, and 100 μl of streptavidin linked to acetylcholinesterase (G4) was deposited in the wells. After 1 h at room temperature, the plates were washed again and 200 μl of Ellman's medium was added to the wells. After a 30-min incubation, the absorbance was measured at 414 nm. The two mice showing the best immune response were selected for Mab production and given a daily intravenous booster injection of 50 μg immunogen for 3 days. Two days after the last boost, hybridoma were produced by fusing spleen cells with NS1 myeloma cells. Hybridoma culture supernatants were screened for antibody production by immunoenzymatic assay. Briefly, hybridoma were distributed on 20 microplates and culture supernatants were tested following Format 1 immunoenzymatic assay (Figure 1). Biotinylated surface proteins of *Candida auris* (#8971) were used for this immunoenzymatic assay. Hybridoma contained in wells giving the highest signals were selected. For the second selection step, hybridoma were tested by immunoenzymatic tests Format 1 and 2 (Figure 1). Biotinylated surface proteins of 3 *Candida auris* strains (#8971, #8977, #8984) and 1 *Candida haemulonii* strain (#8402) were tested separately following Format 1. Four different microplate coatings at 5 × 10^8^ CFU/ml with 3 *Candida auris* strains (#8971, #8977, #8984) and 1 *Candida haemulonii* strain (#8402) were tested following Format 2. Hybridoma contained in wells giving the highest signals for *Candida auris* in any conditions and no signal for *Candida haemulonii* were selected for the next steps. The hybridoma were subsequently cloned by limiting dilutions. Mabs were produced *in vitro* and were purified using protein G affinity chromatography. The purity of the Mabs was then assessed by SDS-PAGE in reducing and non-reducing conditions.

To evaluate the best Mab pairs for use in the LFIA, a combinatorial analysis was carried out with each Mab either as capture or detection antibody, using shaved *Candida* surface proteins or whole *Candida* yeast as target. The colloidal-gold-labeled Mabs were prepared according to NG-Biotech instructions and the strips (0.5 cm width and 4.5 cm length) were prepared as follows. The test strip comprised a sample pad, a nitrocellulose membrane, and an absorption pad, all attached to a backing card. The detection zone comprised immobilized anti-mouse antibodies as a control line and anti-*Candida auris* antibodies as a test line (0.5 and 1 mg/ml in 50 mM potassium phosphate buffer pH 7.4, respectively) dispensed at 1 μl/cm using an automatic dispenser (BioDot Airjet XYZ 3050; BioDot, Irvine, CA, USA). After drying for 30 min at 37°C in an air oven, the absorption pad and the sample pad were stuck to the top and the bottom edges of the membrane, respectively. The membranes were cut into strips of 5 mm width using an automatic programmable cutter (Guillotine Cutting CM4000; BioDot). Shaved Candida surface proteins were mixed in migration buffer to a final concentration of 5 μg/ml supplemented with 0.01% N-ethylmaleimide. Whole yeasts were mixed in migration buffer to a final absorbance of OD_600_ = 1. After 5 min incubation, 100 μl of shaved Candida surface proteins or whole yeast suspensions were mixed with 10 μl of colloidal-gold-labeled Mabs in a 96-well microtiter plate and incubated for an additional 5 min. The LFIA strips were then inserted into the wells with the sample pad in contact with the liquid. After a 15 min migration the results were determined visually.

### 2.5 *Candida auris* LFIA prototype validation on solid media

**Figure 2:**
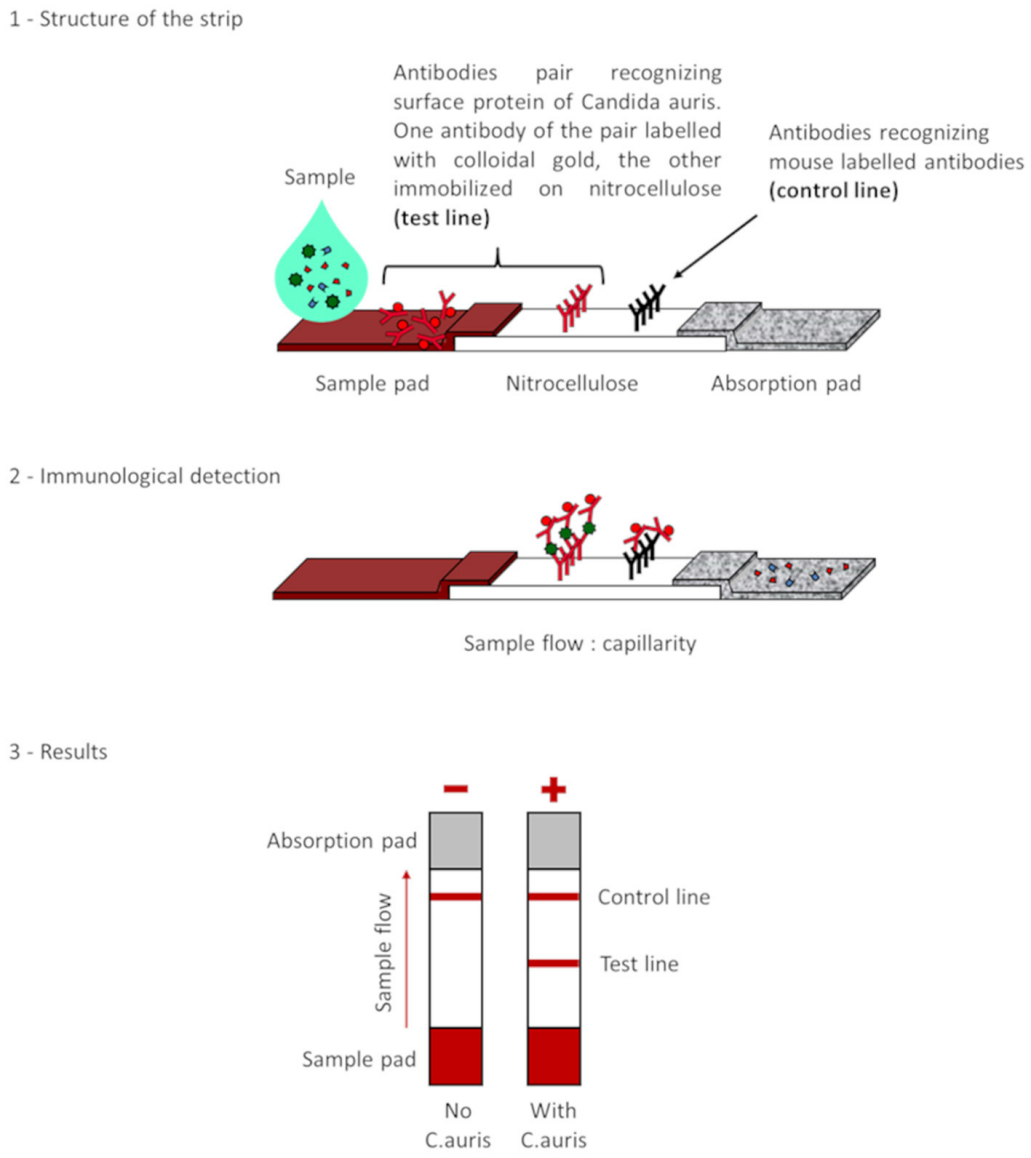
Principle of the lateral flow immunoassay *Candida auris* prototype.

The selected antibodies were produced on a large scale by CEA and provided to NG biotech (Guipry, France) for the development of the *Candida auris* LFIA prototype (Figure 2). Mouse anti-*Candida auris* capture antibody was immobilized on a test line and anti-mouse antibody was immobilized on a control line. The *Candida* strains (Table 1) used for validation of the LFIA prototype were grown for 48 h at 37°C on Sabouraud dextrose, CHROMagar^TM^ *Candida* +, and HardyCHROM^TM^ *Candida* + auris agar plates, with the exception of *Candida dubliniensis, Candida catenulata* and *Candida duobushaemulonii* which required 3, 4, and 5 days of growth, respectively. Using a 1 μL inoculation loop, three colonies were taken and resuspended in 150 μL of the lysis buffer and vortexed. A volume of 150 μL of migration buffer was added and the tube was vortexed again. Finally, 100 μL of the yeast extract was transferred onto the LFIA prototype and allowed to migrate for 15 min. The results were determined visually by monitoring the appearance of a red band on the test line, along with a band corresponding to the internal control.

### 2.6 *Candida auris* LFIA prototype validation on enrichment broth

The LFIA prototype was also evaluated on two different enrichment broths: SSDB manufactured by S2Media^TM^ (0.5% casein digest, 0.5% peptic digest of animal tissue, 2% dulcitol, 10% NaCl, 50 mg/L gentamicin and chloramphenicol) and a second prepared by adding 30 g of Sabouraud-dextrose (Beckton Dickinson), 100 g NaCl (10%) and 50 mg of chloramphenicol into 1 L of demineralized water. The solution was mixed under agitation until complete dissolution and then sterile-filtered using a 0.45 μ filter. Some of the *Candida* strains listed in Table 1 were then inoculated as follows. Each strain was cultured on a Sabouraud dextrose agar plate for 48–120 h at 37°C and colonies were added to sterile physiological water to reach 0.5 McFarland (equivalent to approximately ≈1–5 10^6^ cells/mL). Then, 70 μL of this suspension was spiked into 7 mL of each of the enrichment broths. The inoculated broths were incubated at 40°C under agitation at 250 rpm for a maximum of 72 h with a visual monitoring of turbidity every 24 h. The enrichment broths were then processed as follows on the rapid test every 24 h even if no turbidity was noticed. First, 1 mL of broth was added to a microtube and centrifuged for 1 min at 10,000 g. The supernatant was discarded and the pellet was resuspended into 1 mL of PBS and centrifuged again for 1 min at 10,000 g. The supernatant was discarded and the pellet was processed by adding 150 μL of Lysis buffer^TM^, vortexed, with the addition of 150 μL of migration buffer after a final vortex. Finally, 100 μL of the yeast extract was transferred onto the LFIA prototype, and allowed to migrate for 15 min. The results were visually determined by monitoring the appearance of a red band on the test line, along with a band corresponding to the internal control.

## 3 Results

### 3.1 Selection of *Candida auris* antibodies

After fusion of NS1 and lymphocyte B cells, 104 hybridoma presenting the highest signals were selected with Format 1 (Figure 1) immunoenzymatic assay. Twenty hybridoma presenting the highest signals for *Candida auris* and no signal for *Candida haemulonii* were then selected with Format 1 and 2 (Figure 1) immunoenzymatic assays. After cloning of hybridoma cell lines by limiting dilution, 18 monoclonal antibodies were obtained and successfully purified. Of 37 pairs of antibodies performing on the LFIA format, five pairs with the best LFIA performance were selected for preliminary specificity and sensitivity testing. A final selection based on these two parameters allowed us to integrate the best pair into the LFIA prototype.

### 3.2 *Candida auris* LFIA prototype evaluation on Sabouraud dextrose, CHROMagar^*TM*^ *Candida* +, and HardyCHROM^*TM*^ *Candida* + *auris* agar plates

The specificity and sensitivity results obtained on the LFIA prototype using the selected Mabs are shown in Tables 2, 3, respectively. All the 37 *Candida* non-*auris* strains tested were found to be negative demonstrating a specificity of 100%, and all 12 *Candida auris* strains, covering the four main clades, were detected with a clear unambiguous signal demonstrating a sensitivity of 100%. Results between Sabouraud dextrose, CHROMagar^TM^ *Candida* +, and HardyCHROM^TM^ *Candida* + *auris* agar plates were concordant in terms of sensitivity and specificity. However, a slightly decreased signal was noted on the rapid test for both chromogenic medias, compared to the standard Sabouraud dextrose agar plate.

**Table 2:** Specificity results with various *Candida* non-*auris* isolates grown on Sabouraud, CHROMagar^TM^ *Candida* + and HardyCHROM^TM^ *Candida* + *auris* agar plates with the lateral flow immunoassay prototype using the best selected antibody pair.

**Strain**	**Result on the LFIA prototype (Positive/Negative)**			**Picture**	
	**Sabouraud**	**CHROMagar^TM^ *Candida* +**	**HardyCHROM^TM^ *Candida* + *auris***	**C**	**T**
*Candida albicans (Z006)*	Negative	Negative	Negative	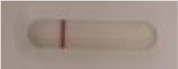	
*Candida glabrata (Z007)*	Negative	Negative	Negative	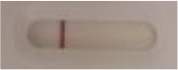	
*Candida krusei (Z009)*	Negative	Negative	Negative	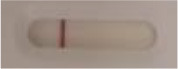	
*Candida parapsilosis (Z011)*	Negative	Negative	Negative	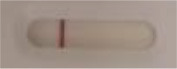	
*Candida tropicalis (Z012)*	Negative	Negative	Negative	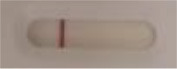	
*Candida guilliermondii (Z008)*	Negative	Negative	Negative	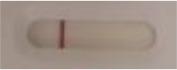	
*Candida lusitaniae (Z010)*	Negative	Negative	Negative	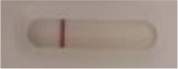	
*Candida sojae (Z128)*	Negative	Negative	Negative	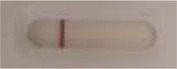	
*Candida kefyr (Z110)*	Negative	Negative	Negative	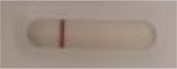	
*Candida dubliniensis (Z145)*	Negative	Negative	Negative	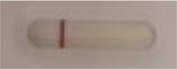	
*Candida catenulata (Z253)*	Negative	Negative	Negative	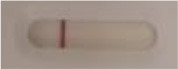	
*Candida albicans (18804)*	Negative	Negative	Negative	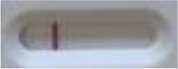	
*Candida tropicalis (1369)*	Negative	Negative	Negative	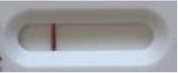	
*Candida haemulonii (8402)*	Negative	Negative	Negative	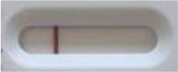	
*Candida krusei (34135)*	Negative	Negative	Negative	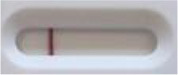	
*Candida tropicalis (66029)*	Negative	Negative	Negative	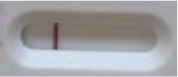	
*Candida albicans (64124)*	Negative	Negative	Negative	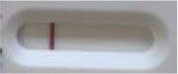	
*Candida albicans (14058)*	Negative	Negative	Negative	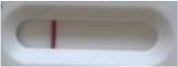	
*Candida krusei (14243)*	Negative	Negative	Negative	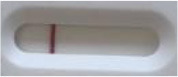	
*Candida parapsilosis (22019)*	Negative	Negative	Negative	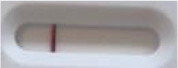	
*Candida glabrata (66032)*	Negative	Negative	Negative	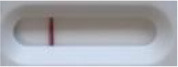	
*Candida tropicalis (13803)*	Negative	Negative	Negative	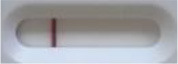	
*Candida glabrata (2001)*	Negative	Negative	Negative	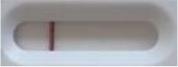	
*Candida glabrata (15126)*	Negative	Negative	Negative	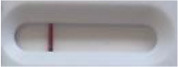	
*Candida albicans (NR-29342)*	Negative	Negative	Negative	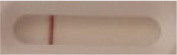	
*Candida albicans (NR-29343)*	Negative	Negative	Negative	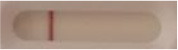	
*Candida albicans (NR-29344)*	Negative	Negative	Negative	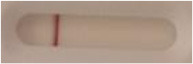	
*Candida albicans (NR-29452)*	Negative	Negative	Negative	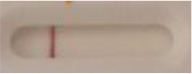	
*Candida albicans (NR-29453)*	Negative	Negative	Negative	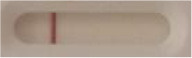	
*Candida glabrata (HM-1123)*	Negative	Negative	Negative	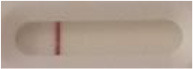	
*Candida krusei (HM-1122)*	Negative	Negative	Negative	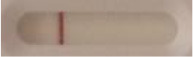	
*Candida tropicalis (HM-1124)*	Negative	Negative	Negative	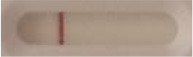	
*Candida duobushaemulonii (105996)*	Negative	Negative	Negative	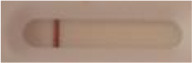	
*Candida duobushaemulonii (105997)*	Negative	Negative	Negative	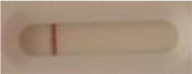	
*Candida duobushaemulonii (105999)*	Negative	Negative	Negative	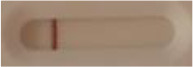	
*Candida haemulonii (70624)*	Negative	Negative	Negative	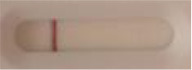	
*Candida haemulonii (105998)*	Negative	Negative	Negative	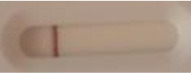	

Isolates were collected with a 1 μL loop by taking 3 colonies and processed using the kit protocol. Results were interpreted visually at 15 min. As all results were negative only the pictures taken with the Sabouraud agar plate are shown. C, Control line; T, Test line.

**Table 3:** Sensitivity results with various *Candida auris* isolates grown on Sabouraud, CHROMagar^TM^ *Candida* + or HardyCHROM^TM^ *Candida* + *auris* agar plates with the lateral flow immunoassay prototype using the best selected antibody pair.

**Strain**	**Result on the LFIA prototype (Positive/Negative)**								
	**Sabouraud**	**Picture**		**CHROMagar^TM^ *Candida* +**	**Picture**		**HardyCHROM^TM^ *Candida* + *auris***	**Picture**	
		**C**	**T**		**C**	**T**		**C**	**T**
*Candida auris* (Z485)	Positive	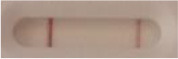		Positive	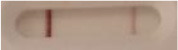		Positive	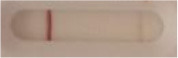	
*Candida auris* (105991/CDC AR-Bank 0386)	Positive	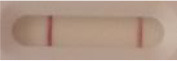		Positive	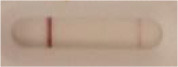		Positive	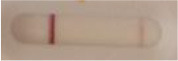	
*Candida auris* (105989/ CDC AR-Bank 0384)	Positive	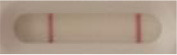		Positive	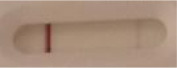		Positive	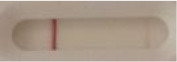	
*Candida auris* (105990/ CDC AR-Bank 0385)	Positive	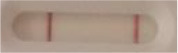		Positive	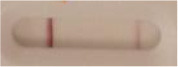		Positive	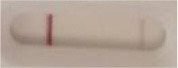	
*Candida auris* (105988/ CDC AR-Bank 0383)	Positive	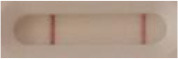		Positive	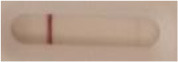		Positive	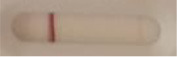	
*Candida auris* (105987/ CDC AR-Bank 0382)	Positive	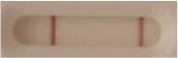		Positive	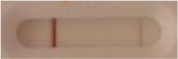		Positive	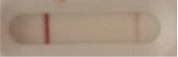	
*Candida auris* (105992/ CDC AR-Bank 0387)	Positive	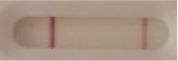		Positive	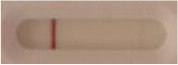		Positive	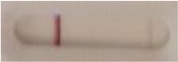	
*Candida auris* (21092)	Positive	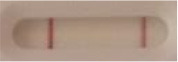		Positive	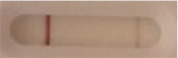		Positive	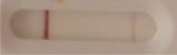	
*Candida auris* (NR-52714)	Positive	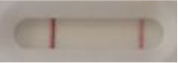		Positive	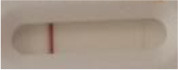		Positive	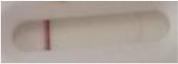	
*Candida auris* (NR-52715)	Positive	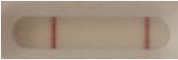		Positive	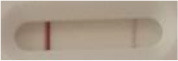		Positive	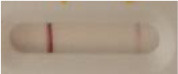	
*Candida auris* (NR-52716)	Positive	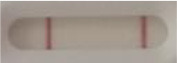		Positive	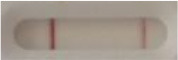		Positive	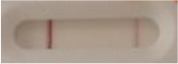	
*Candida auris* (NR-52717)	Positive	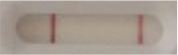		Positive	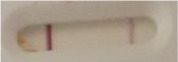		Positive	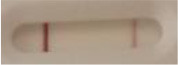	

Isolates were collected by taking 3 colonies with 1 μL loop and processed using the kit protocol. Results were interpreted visually at 15 min. The pictures represent the results at the reading time (left line, control line; right line, test line if any).

### 3.3 *Candida auris* LFIA prototype evaluation on enrichment broth

Most of the *Candida* non-*auris* strains did not grow in the broths used, with the exception of *Candida guilliermondii, Candida glabrata, Candida duobushaemulonii* and/or *Candida parapsilosis*. In contrast, all *Candida auris* strains grew in both media. However, significant differences were seen between the two broths. The *Candida auris* strains grew more quickly on the dextrose-based broth with 83.3% (Table 4) culture positive compared to 41.7% (Table 5) culture positive with SSDB after 24 h incubation. With both broths, 100% culture positive was reached after 48 h incubation. When cultured on dextrose-based media, all *Candida auris* species found positive at 48 h were also found positive on the rapid test at 24 h. However, with SSDB, none of the *Candida auris* species found positive from culture at 48 h were positive on the rapid test, which may be explained by the weak turbidity obtained at 24 h.

**Table 4:** Number of positive culture and rapid tests from various *Candida auris* and non*-auris* isolates grown on dextrose-sabouraud enrichment broth containing 10% NaCl and 50 mg/L chloramphenicol at 40°C for up to 72 h.

**Strain**	**Number of positive culture and rapid tests according to time of culture**					
	**24 h**		**48 h**		**72 h**	
	**Positive culture**	**Positive rapid test**	**Positive culture**	**Positive rapid test**	**Positive culture**	**Positive rapid test**
None (*n* = 1)	0	0	0	0	0	0
*Candida* non-*auris* (*n* = 23)	0 (0%)	0 (0%)	3 (13.0%)	0 (0%)	3 (13.0%)	0 (0%)
*albicans* (*n* = 6)	0	0	0	0	0	0
*krusei* (*n* = 2)	0	0	0	0	0	0
*guilliermondii* (*n* = 1)	0	0	1	0	1	0
*dubliniensis* (*n* = 1)	0	0	0	0	0	0
*tropicalis* (*n* = 2)	0	0	0	0	0	0
*parapsilosis* (*n* = 1)	0	0	0	0	0	0
*glabrata* (*n* = 2)	0	0	2	0	2	0
*kefyr* (*n* = 1)	0	0	0	0	0	0
*lusitaniae* (*n* = 1)	0	0	0	0	0	0
*sojae* (*n* = 1)	0	0	0	0	0	0
*duobushaemulonii* (*n* = 3)	0	0	0	0	0	0
*haemulonii* (*n* = 2)	0	0	0	0	0	0
*Candida auris* (*n* = 12)	9 (83.3%)	9 (83.3%)	12 (100%)	12 (100%)	12 (100%)	12 (100%)

A culture was considered positive once a turbidity was noticed. Broths were processed on the rapid test by two successive centrifugations for 1 min at 10,000 g to recover and wash the pellet which was then assayed using the kit protocol. Results were interpreted visually at 15 min.

**Table 5:** Number of positive culture and rapid tests from various *Candida auris* and non*-auris* isolates grown on Salt Sabouraud Dulcitol Broth (SSDB) containing 50 mg/L chloramphenicol and gentamicin at 40°C for up to 72 h.

**Strain**	**Number of positive culture and rapid tests according to time of culture**					
	**24 h**		**48 h**		**72 h**	
	**Positive culture**	**Positive rapid test**	**Positive culture**	**Positive rapid test**	**Positive culture**	**Positive rapid test**
None (*n* = 1)	0	0	0	0	0	0
*Candida* non-*auris* (*n* = 23)	0 (0%)	0 (0%)	3 (13.0 %)	0 (0%)	4 (17.4%)	0 (0%)
*albicans* (*n* = 6)	0	0	0	0	0	0
*krusei* (*n* = 2)	0	0	0	0	0	0
*giulliermondii* (*n* = 1)	0	0	1	0	1	0
*dubliniensis* (*n* = 1)	0	0	0	0	0	0
*tropicalis* (*n* = 2)	0	0	0	0	0	0
*parapsilosis* (*n* = 1)	0	0	1	0	1	0
*glabrata* (*n* = 2)	0	0	0	0	1	0
*kefyr* (*n* = 1)	0	0	0	0	0	0
*lusitaniae* (*n* = 1)	0	0	0	0	0	0
*sojae* (*n* = 1)	0	0	0	0	0	0
*duobushaemulonii* (*n* = 3)	0	0	1	0	1	0
*haemulonii* (*n* = 2)	0	0	0	0	0	0
*Candida auris* (n = 12)	5 (41.7%)	0 (0%)	12 (100%)	10 (83.3%)	12 (100%)	12 (100%)

A culture was considered positive once a turbidity was noticed. Broths were processed on the rapid test by two successive centrifugations for 1 min at 10,000 g to recover and wash the pellet which was then assayed using the kit protocol. Results were interpreted visually at 15 min.

## 4 Discussion

The aims of this study were 2-fold: (i) to obtain highly specific Mabs for *Candida auris* surface proteins; and (ii) to design and validate the performance of a LFIA prototype for rapid identification of *Candida auris* from colonies grown on agar plates or directly from enrichment broth. Among 104 pre-selected hybridoma, selection of the best antibody pair for use in the LFIA prototype achieved high identification accuracy. Indeed, this rapid test was able to detect 100% of the 12 *Candida auris* strains tested, covering the four mains clades, as well as being 100% specific for 37 other *Candida* species, including those most encountered in clinical settings (Yapar, 2014). All results were concordant when the strains were grown on a regular sabouraud-dextrose agar plate or on CHROMagar^TM^ *Candida* Plus and HardyCHROM^TM^ *Candida* + auris media, although a slightly reduced signal was noted with the rapid test on the chromogenic media. Furthermore, the LFIA prototype was able to detect all the *Candida auris* strains directly from turbid enrichment broths using a simple protocol for yeast concentration by centrifugation with excellent accuracy. In our study, the use of SSDB did not provide any advantages over a dextrose-based broth with chloramphenicol and salt. In both cases, some *Candida* non-*auris* species were still able to grow (*Candida glabrata, Candida guilliermondii, Candida duobushaemulonii*, and/or *Candida parapsilosis*) indicating that the SSDB was not more selective. On the contrary, the use of SSDB resulted in a growth decrease for some *Candida auris* strains, lengthening the time to result.

Although this LFIA prototype shows very promising results, further data are required such as determining its ability to detect the fifth clade of *Candida auris* that has recently emerged (Chow et al., 2019), as well as the specificity of the antibodies against other rare *Candida* species or microorganisms such as *Candida famata*, which can be misidentified with other methods (Keighley et al., 2021).

Mycological culture remains central to the diagnostic approach with *Candida auris*. However, most of the commercially available agar plates are unable to differentiate *Candida auris* from other species (Keighley et al., 2021). Nonetheless, a recent study showed that CHROMagar^TM^ *Candida* Plus could accurately distinguish *Candida auris* from other species, but the ambiguous colors sometimes found with chromogenic media can lead to uncertainty regarding the identification (Borman et al., 2021; Mulet Bayona et al., 2022). Furthermore, false positive results have been seen with *Candida pseudohaemulonii* and *Candida vulturna* (de Jong et al., 2021). Consequently, a confirmatory test is always required.

Non-culture-based methods, such as phenotypic and biochemical identification, show many limitations when differentiating *Candida auris* from other species (Keighley et al., 2021). However, matrix-assisted laser desorption ionization time-of-flight (MALDI-TOF) mass spectrometry has proven to be accurate and reliable at identifying *Candida auris* species as well as characterizing the antifungal susceptibility profile (Keighley et al., 2021; Abdolrasouli and Fraser, 2022). Although the time to results is short and the cost of analysis is affordable (Dhiman et al., 2011), this assay still requires an expensive analyzer and trained technicians thus limiting broad availability of the method. Molecular amplification-based methods, such as Polymerase Chain Reaction (PCR) or Loop-Mediated Amplification Method (LAMP), are also powerful tools and accurate for *Candida auris* identification (Yamamoto et al., 2018; Alvarado et al., 2021; Mulet Bayona et al., 2021; Zhang et al., 2023). However, commercially available kits are mainly used in positive blood cultures or other direct samples considering their high cost (Lockhart et al., 2022). Finally, DNA sequencing is also a powerful tool for *Candida auris* identification, but is complex and expensive and consequently not available in most clinical microbiology laboratories (Lockhart et al., 2022). In the past decade, LFIA rapid tests have emerged as important and reliable tools in the field of clinical microbiology, particularly in the detection of AMR (Boutal et al., 2022). The newly-developed LFIA prototype overcomes many limitations of currently available methods for *Candida auris* identification and is affordable, does not require expensive equipment, is easy to use, can be stored long term at room temperature, and provides an unambiguous interpretation. This will allow its broad use in clinical laboratories in low as well as high income countries. In addition, the ability to provide an identification directly from a turbid enrichment broth reduces the time to final identification by around 48 h compared with surveillance samples enrichment followed by isolation on solid media. This allow to save precious time when it comes to patient isolation to limit the spread of *Candida auris* in hospital settings.

## 5 Conclusion

In conclusion, we have developed a new LFIA prototype for the rapid identification of *Candida auris* on isolates grown on agar plates or directly from positive enrichment broths used in surveillance. We anticipate that this powerful new tool will help fight the dissemination of the emerging threat of *Candida auris* in clinical settings. The strength of our study is that the LFIA prototype reached 100% specificity and sensitivity on a highly diverse and clinically relevant *Candida auris* and *Candida* non-*auris* panel, including species often falsely mixed-up with *Candida auris*. The weakness of our study is that the LFIA prototype was evaluated on a relatively low number of strains and the detection from enrichment broth performed on contrived specimens only. Therefore, validation of the LFIA prototype in clinical setting represents the next step.

## Data availability statement

The original contributions presented in the study are included in the article/supplementary material, further inquiries can be directed to the corresponding author.

## Author contributions

AC: Writing – original draft, Validation, Investigation, Funding acquisition, Conceptualization. AA: Writing – original draft, Validation, Investigation, Conceptualization. A-SH: Writing – original draft, Methodology, Investigation. MP: Writing – original draft, Methodology, Investigation. LN: Writing – original draft, Methodology, Investigation. SS: Writing – original draft, Supervision, Funding acquisition. HV: Funding acquisition, Writing – review & editing, Supervision, Project administration.
